# Chemotactic Chemokines Are Important in the Pathogenesis of Irritable Bowel Syndrome

**DOI:** 10.1371/journal.pone.0093144

**Published:** 2014-03-25

**Authors:** Charles Darkoh, Latoya Comer, Getie Zewdie, Stephen Harold, Ned Snyder, Herbert L. DuPont

**Affiliations:** 1 The University of Texas School of Public Health, Division of Epidemiology, Human Genetics, and Environmental Sciences, Center for Infectious Diseases, Houston, Texas, United States of America; 2 The University of Texas Medical School, Houston, Texas, United States of America; 3 Kelsey Research Foundation, Houston, Texas, United States of America; 4 Kelsey Seybold Clinic, Houston, Texas, United States of America; 5 St. Luke's Medical Center, Internal Medicine, Houston, Texas, United States of America; 6 Department of Medicine, Baylor College of Medicine, Houston, Texas, United States of America; University of Texas School of Public Health, United States of America

## Abstract

Irritable bowel syndrome (IBS) is one of the most frequently diagnosed disorders, affecting about 20% of the general population in Western countries. This syndrome poses an enormous socio-economic burden, impairs the quality of life substantially, and increases healthcare costs. IBS can be classified as either idiopathic (ID-IBS) with unknown etiology or post-infectious (PI-IBS), which develops after a bout of acute diarrhea or gastroenteritis. Little is known about the immunopathogenesis of these two forms of IBS. We evaluated various biomarkers in clinical samples of ID-IBS and PI-IBS patients with the goal to test the hypothesis that the immunologic presentations of these forms of IBS are similar, despite their apparent different etiologic origins. Sera and stool samples from PI-IBS, ID-IBS, and healthy volunteers were analyzed for relative amounts of 36 different biomarkers using the Proteome Profiler Human Cytokine Array Panel A Kit and quantitative ELISA. Our results demonstrated significantly high levels of chemotactic chemokines monocytes chemotactic protein-1 (CCL2) [p-value  = 0.003], macrophage inflammatory protein-1β (CCL4) [p-value  = 0.010], and CXCL16 (p-value 0.001) in the sera and stools of both ID-IBS and PI-IBS patients. Furthermore, pro-inflammatory cytokines (IFN-γ, IL-1β, and TNF-α) were significantly higher in IBS patients. Anti-inflammatory cytokines (IL-10, IL-4, and IL-13) were variable except IL-10, which was significantly higher in the healthy volunteers than the IBS patients. Remarkably, the amounts and expression pattern of these biomarkers were not significantly different between ID-IBS and PI-IBS. Thus, ID-IBS and PI-IBS present similar immunologic and clinical phenotypes, in spite of their different etiologic origins.

## Introduction

Irritable bowel syndrome (IBS), which affects about 20% of the general population [1] is associated with chronic, relapsing, and idiopathic inflammation of the gastrointestinal (GI) tract, thought to be a consequence of aberrant activation of the innate and adaptive immune responses [2], [3], [4]. The underlying inflammatory process leads to increased enterochromaffin cells, T-lymphocytes, intestinal permeability, colonic transit time, and a variety of immunologic abnormalities in the GI tract. IBS affects an estimated 1.4 to 2.2 million persons annually in the USA and Europe [3]. IBS patients may be classified as either idiopathic (ID-IBS) with an unknown cause or post-infectious (PI-IBS), which develops after a bout of acute diarrhea, gastroenteritis or bacterial infection [5]. Several enteropathogens have been implicated in the pathogenesis of PI-IBS, including *Shigella* [6], Shiga toxin-producing *E. coli*, *Campylobacter jejuni* [7], *Salmonella* [8], and enterotoxigenic *E. coli* [9]. Risk factors for developing IBS include the virulence of the infecting microbe, the severity of the bout of diarrhea or gastroenteritis, host demographics (e.g. age and sex), pre-existing anxiety and depression [7], [10], persistent inflammation subsequent to infection, and alterations in gut physiology.

ID-IBS and PI-IBS present similar symptoms such as chronic colonic inflammation, delayed motility, abdominal pain and distension, irregular bowel movement, and bloating [11], [12]. However, how these two different forms of IBS with different etiologic origins produce similar disease symptoms and how different inflammatory markers may predispose patients to IBS are unknown. We hypothesized that PI-IBS and ID-IBS result from different etiologic pathways and converge during the immunopathologic process to produce similar disease symptoms. Here, we compared the various pro-inflammatory and anti-inflammatory cytokines, chemokines, and acute proteins in healthy and IBS-patients to gain insight into how these biomarkers may contribute to the disease process. We report for the first time that the concentrations of chemotactic chemokines MCP-1 (CCL2), MIP-1β (CCL4), and CXCL16 are significantly higher in the sera and stools of IBS patients compared to healthy volunteers.

## Materials and Methods

### Study Population

Patients (≥18 years) diagnosed with IBS at a large medical clinic in Houston, Texas were enrolled in this study. The definition of IBS was based on the Rome II criteria [13], [14]. The patients were grouped into idiopathic (ID) and post-infectious (PI) IBS. PI-IBS was defined as patients who meet the Rome II criteria with the onset of their chronic intestinal problem preceded by a bout of acute diarrhea or gastroenteritis [15]. All other patients that met the Rome II criteria for IBS were considered ID-IBS. The healthy volunteers were enrolled from subjects who visited the clinic for annual physical evaluations. The healthy volunteers were matched (by percentage enrolled) for race and gender to IBS cases. IBS patients with underlying conditions that are known to influence gastrointestinal function, including diabetes, pregnancy, breast feeding, cirrhosis, celiac disease, and inflammatory bowel disease were excluded from the study. A total of 44 ID-IBS, 16 PI-IBS, and 40 healthy volunteers were enrolled for the study.

### Blood Sample Collection and Processing

The protocol used in this study was approved by the Institutional Review Boards of The University of Texas Health Science Center at Houston and St. Luke's Medical Center (Houston, Texas). All of the participating patients or their legal guardians provided written informed consent upon admission to the clinic. Blood samples were collected directly into Vacutainer CPT tubes (BD, Franklin Lakes, NJ) containing sodium citrate. The serum was separated from the polymorphonuclear cells based on the instructions provided by the manufacturer. The samples were stored at −80°C until tested.

### Cytokine Assays

Sera from 44 ID-IBS, 16 PI-IBS, and 40 healthy volunteers were initially evaluated for the presence and relative amounts of 36 different biomarkers using the Proteome Profiler Human Cytokine Array Panel A Kit (R&D Systems, Minneapolis, MN). This assay simultaneously detects 36 different biomarkers directly in a biological sample. A list of all the 36 cytokines/chemokines tested and their reported roles in inflammation and immune response is shown in Table S1 in File S1. Each serum sample (250 μL) was thoroughly suspended in a 1.25 ml of Array Buffer 5, incubated at room temperature for 15 min, and centrifuged for 5 min at 5,000×g. The supernatant (1 ml each) was added to a cocktail of biotinylated antibodies and incubated at room temperature for 1 hr. The sample-antibody mixture was subsequently incubated at 4°C for 19 hrs with a membrane embedded with antibodies specific to each of the 36 different biomarkers analyzed. Following a washing step, 3 ml of a 1∶1000 dilution of secondary antibody conjugated with streptavidin-HRP was added to each membrane and incubated at room temperature for 45 min. For detection, the membranes were probed with the Pierce ECL Western Blotting Substrate (Thermo Scientific, Rockford, IL) and exposed to an X-ray film. The exposed film was processed using SRX-101A Medical Film Processor (Konica Minolta). The intensity of each blot (band) representing the amount of each biomarker present were measured using the ImageJ software (National Institutes of Health, Bethesda, MD) and expressed as pixel densities for comparative analysis.

All the images from the 100 arrays were normalized by subtracting the background and inverted to eliminate the background differences. To measure the pixel density, a fixed size rectangular box was generated around each dot blot/band and the pixel density was measured. The same sized rectangular box was used for all the bands in all the 100 arrays performed. For analysis, the pixel densities of the negative control on each array were subtracted from the pixel densities obtained from each band on the array. The data was further converted and normalized into fold change in expression by dividing the pixel densities of each band by the average pixel densities of the Streptavidin-HRP Reference Spots located at the three corners of each array.

### Quantitative ELISA Assays

The serum and fecal concentrations of the biomarkers that were initially established to be important in the pathogenesis of IBS (MCP-1, MIP-1β, TNF-α, IFN-γ, IL-1β, IL-10, IL-4, IL-13, and CXCL16), based on the initial data from the Proteome Profiler Human Cytokine Array assay, were determined by quantitative ELISA (R&D Systems, Minneapolis, MN). The instructions provided by the manufacturer were followed in determining the serum concentrations of each of these biomarkers. The sensitivity and the assay range, respectively, of the ELISA test for each of the biomarkers tested are as follows: MCP-1 (10 pg/mL, 31.2–2,000 pg/mL); MIP1-β (11 pg/mL, 15.6–1,000 pg/mL); IFN-γ (8 pg/mL, 15.6–1,000 pg/mL); TNF-α (5.5 pg/mL, 15.6–1,000 pg/mL); IL-1β (1 pg/mL, 3.9–250 pg/mL); IL-10 (3.9 pg/mL, 7.8–500 pg/mL); CXCL16 (0.017 ng/mL, 0.156–10 ng/mL).

For the fecal concentrations, stool samples (200 mg) from 20 healthy volunteers, 20 ID-IBS, and 10 PI-IBS were suspended in 500 μL of PBS and centrifuged at 10,000×g for 10 min to remove the debris and solid materials in present. The supernatant fluids were tested for the biomarkers listed above according to the manufacturer's instructions.

### Statistical Analysis

The data were analyzed by logistic regression. Statistical analysis was performed using the statistical packages R version 2.12.1 (R Foundation for Statistical Computing, Vienna, Austria) and Stata Statistical Software Release 12.1 (StataCorp LP, College Station, TX). Mann-Whitney two-tailed non-parametric test of significance and One-Way ANOVA were performed using GraphPad Prism version 6 for Windows (GraphPad Software, San Diego, CA).

## Results

### Characteristics of the Study Population

To uncover the biomarkers expressed by ID-IBS and PI-IBS patients during episodes of the syndrome, we evaluated sera and fecal samples for different biomarkers present. The study population included 44 ID-IBS, 16 PI-IBS, and 40 healthy volunteers aged between 24 and 86 years (Table 1). The average ages among the study groups were 54, 50, and 53 years for ID-IBS, PI-IBS, and healthy volunteers, respectively. The majority of the patients enrolled were from three major ethnicities: White/Caucasians, Black/African-Americans, and Hispanics/Latinos. Patients with underlying conditions that are known to influence gastrointestinal functions such as diabetes, pregnancy, breast feeding, cirrhosis, and celiac disease were excluded from the study. No other co-morbidities were noted.

**Table 1:** The characteristics of the study population.

Characteristics	PI-IBS (n = 16)	ID-IBS (n = 44)	Healthy Controls (n = 40)
**Average age (range)**	50 (28–66)	54 (24–79)	53 (25–86)
**Ethnicity**			
**Asian**	1	0	0
**B/AA**	5	17	18
**H/L**	7	10	9
**W/C**	3	17	13
**Gender**			
**Female**	13	36	23
**Male**	3	8	17

IBS patients with underlying conditions that are known to influence gastrointestinal function other than IBS including diabetes, pregnancy, breast feeding, cirrhosis, celiac disease, and inflammatory bowel disease were excluded from the study.

PI-IBS, post-infectious IBS; ID-IBS, idiopathic IBS; B/AA, Black/African-America; H/L, Hispanic/Latino; W/C, White/Caucasian.

### IBS Patients Express High Levels of Monocytes Chemotactic Protein-1 (MCP-1) and Macrophage Inflammatory Protein-1β (MIP-1β)

**Figure 1:**
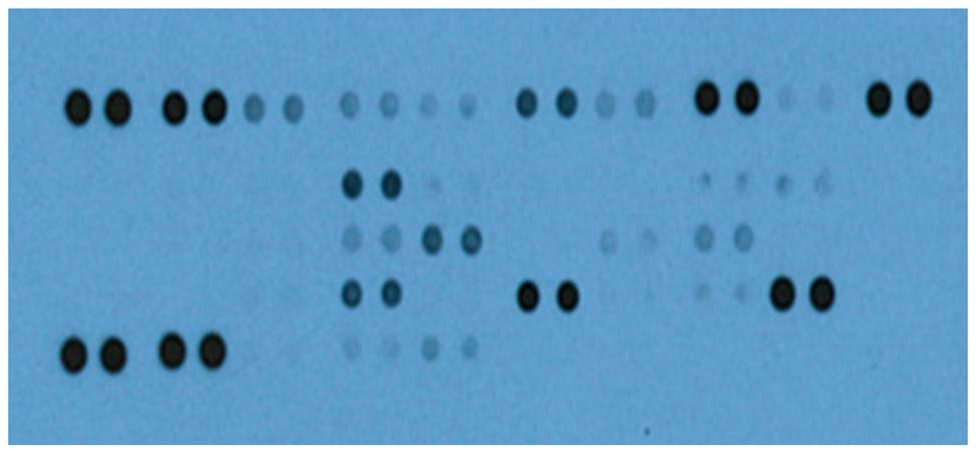
A representative image of the various biomarkers detected in the sera of IBS patients and healthy volunteers. Each pair of horizontal blots (bands) represent a different biomarker present in the serum, whereas the intensities of the blots characterize the amount of the respective biomarker. Sera (250 μL) from 60 IBS patients (44 idiopathic IBS and 16 post-infectious IBS) and 40 healthy volunteers were evaluated for the presence of 36 different biomarkers using the Proteome Profiler Human Cytokine Array Panel A Kit (R&D Systems, Minneapolis, MN). Samples were incubated with a membrane embedded with biotinylated antibodies that are specific for each of the 36 biomarkers analyzed. For detection, the membranes were probed with the Pierce ECL Western Blotting Substrate (Thermo Scientific, Rockford, IL) and exposed to an X-ray film, following incubation with a secondary antibody labelled with streptavidin-horse radish peroxidase. The exposed film was processed using SRX-101A Medical Film Processor (Konica Minolta). Intensities of the blots were determined and expressed as pixel densities using ImageJ (National Institutes of Health, Bethesda, MD).

We initially tested sera from all the 100 study subjects for the presence and relative amounts of 36 major different biomarkers using the Proteome Profiler Human Cytokine Array Panel A Kit (R&D Systems, Minneapolis, MN). This Western blot-like assay simultaneously detects the relative amounts of 36 different biomarkers present in a sample. Following chemiluminescent detection, the intensity of the blots (bands) from each biomarker corresponds to the amount of that biomarker present in the sample. A representative image of the blots is shown in Figure 1. Bands of different intensities were observed both in individual serum and between sera from different individuals. Some of the 36 different biomarkers tested for were undetected. The number of biomarkers detected was different between the groups of sera examined. The intensities of the blots were empirically determined and represented as pixel densities for each sample using the ImageJ software (National Institutes of Health, Bethesda, MD).

**Figure 2:**
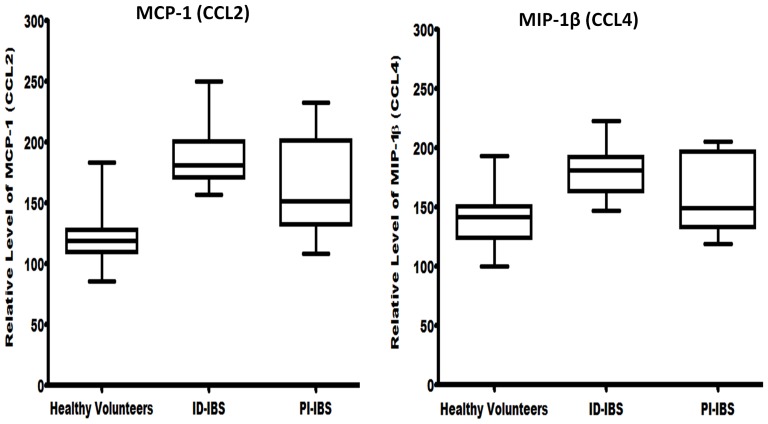
Sera from IBS patients show high levels of monocytes chemotactic protein-1 (MCP-1) and macrophage inflammatory protein-1β (MIP-1β). Sera (250 μL) from IBS patients (44 idiopathic IBS, 16 post-infectious IBS, and 40 healthy volunteers) were analyzed for the presence of 36 biomarkers using the Proteome Profiler Human Cytokine Array Panel A kit (R&D Systems, Minneapolis, MN). Following chemiluminescent detection and processing, the intensities of the blots were determined using ImageJ (National Institutes of Health, Bethesda, MD) and expressed as pixel density. Logistic regression analysis indicated that the amounts of MCP-1 and MIP-1β in the sera were significantly higher in both ID-IBS and PI-IBS (p-values 0.003 and 0.010, respectively) when compared to that of the healthy volunteers. The odds ratios of association between IBS and overexpression of MCP-1 and MIP-1β were 1.054 (95% CI: 1.011–1.100), 1.021 (95% CI: 1.048–1.061), respectively.

To identify biomarkers that were significantly present among the groups, the pixel densities from all the blots were analyzed using the Hosmer and Lemeshow model selection for logistic regression [16], [17]. The amounts of MCP-1 and MIP-1β in the sera were found to be significantly higher in both ID-IBS and PI-IBS (p-values 0.003 and 0.010, respectively), when compared to that of the healthy volunteers (Fig. 2). Remarkably, the amounts of these chemokines in the sera of ID-IBS patients were not significantly different from that of the PI-IBS. The odds ratios of association between IBS and overexpression of MCP-1 and MIP-1β were 1.054 (95% CI: 1.011–1.100), 1.021 (95% CI: 1.048–1.061), respectively. Moreover, these chemokines were detected in a higher proportion of the IBS patients (98% for ID-IBS; 96% for PI-IBS) compared to the healthy volunteers (53%), suggesting that MCP-1 and MIP-1β may play a significant role in the immunopathogenesis of IBS.

**Figure 3:**
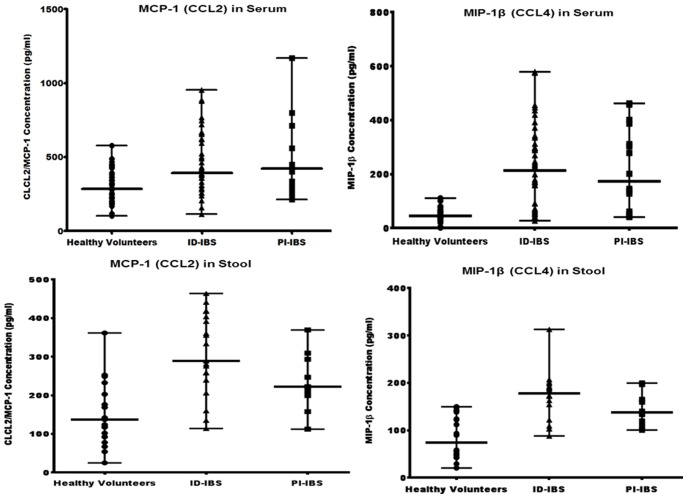
The sera and fecal concentrations of MCP-1 and MIP-1β in IBS patients are significantly higher than healthy volunteers. Sera and stool samples from IBS patients were examined for the concentrations of MCP-1 and MIP-1β by quantitative ELISA (R&D Systems, Minneapolis, MN). Mann Whitney test showed significant differences between the medians of the healthy volunteers versus ID-IBS (P<0.001) and PI-IBS (P<0.0048) for MCP-1; ID-IBS (0.0001), PI-IBS (0.001) for MIP-1β. The medians of ID-IBS versus PI-IBS were not significantly different (P = 0.9267 for MCP-1; P = 0.5308 for MIP-1β). Error bars indicate median values with range.

To validate the preceding findings made from the Proteome Profiler Human Cytokine Array analysis, sera and fecal concentrations of MCP-1 and MIP-1β were determined by quantitative ELISA (R&D Systems, Minneapolis, MN). Both sera and stools from IBS patients contained significantly higher concentrations of MCP-1 and MIP-1β compared to that of the healthy volunteers (Fig. 3). Interestingly, both sera and fecal concentrations of these chemokines from ID-IBS patients were not significantly different from that of PI-IBS patients. The average sera concentrations of MCP-1 and MIP-1β were 295 pg/ml and 47 pg/ml, respectively (for healthy volunteers), 454 pg/ml and 220 pg/ml, respectively (for ID-IBS), 467 pg/ml and 216.2 pg/ml, respectively (for PI-IBS). Furthermore, the average fecal concentrations of MCP-1 and MIP-1β were 149.6 pg/ml and 85.6 pg/ml, respectively (healthy volunteers), 305 pg/ml and 170.4 pg/ml, respectively (ID-IBS), 235.9 pg/ml and 145.9 pg/ml, respectively (PI-IBS). These quantitative ELISA data were consistent with, and supported the data obtained from the Proteome Profiler Human Cytokine Array assay and highlight the importance of MCP-1 and MIP-1β in the pathogenesis of IBS.

### Serum and Fecal Concentrations of Pro-inflammatory Cytokines are Significantly Higher in IBS Patients than Healthy Volunteers

**Figure 4:**
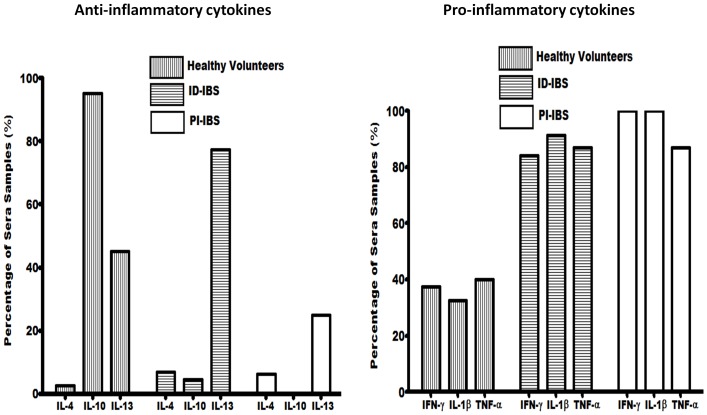
Comparison of the proportion of sera samples with detectable anti-inflammatory cytokines (IL-4, IL-10, and IL-13) and pro-inflammatory cytokines (IFN-γ, IL-1β, and TNF-α). The proportion of sera with detectable anti-inflammatory and pro-inflammatory cytokines were determined following the analysis of sera from 44 ID-IBS, 16 PI-IBS, and 40 healthy volunteers using the Proteome Profiler Human Cytokine Array Panel A kit (R&D Systems, Minneapolis, MN).

**Figure 5:**
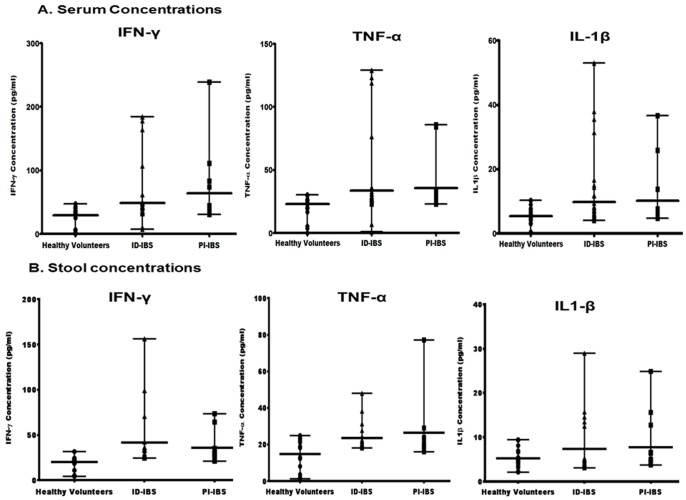
The sera and stools of IBS patients show significantly high levels of pro-inflammatory cytokines (IFN-γ, TNF-α, and IL-1β). Sera and stools from IBS patients and healthy volunteers were analyzed for the concentrations of IFN-γ, TNF-α, and IL-1β by quantitative ELISA (R&D Systems, Minneapolis, MN). Mann Whitney test showed significant difference between the medians of the healthy volunteers versus ID-IBS (P<0.0010) and PI-IBS (P<0.0010) for IFN-γ; ID-IBS (p<0.0001), PI-IBS (p<0.0002) for TNF-α, ID-IBS (P<0.0241) and PI-IBS (P<0.0330) for IL-1β. The medians of ID-IBS versus PI-IBS were not significantly different (P = 0.1618 for IFN-γ; P = 0.4958 for TNF-α; and P = 0.4135 for IL-1β). Error bars indicate median values with range.

MCP-1 and MIP-1β chemokines regulate the migration and recruitment of leukocytes to inflammatory regions. To investigate whether increased levels of these chemokines corresponds to increased levels of inflammatory cytokines, pro-inflammatory and anti-inflammatory cytokines were examined in the sera and stools. Three pro-inflammatory cytokines IFN-γ, IL-1β, and TNF-α were detected in significantly higher number of IBS patients compared to the healthy volunteers (Fig. 4A–B). The average concentrations of IFN-γ, IL-1β, and TNF-α in the sera were 49, 9.8, 34 pg/ml, respectively for ID-IBS, and 64, 10.2, 36 pg/ml, respectively for PI-IBS (Fig. 5). These were significantly higher than that of the healthy volunteers, 29, 5.3, 22.8 pg/ml, respectively. Furthermore, the fecal concentrations of these cytokines were also higher in the IBS patients versus the healthy volunteers (Supplement S2 in File S1). Interestingly, these pro-inflammatory cytokines were not significantly different between ID-IBS and PI-IBS.

The proportion of anti-inflammatory cytokines detected varied between the healthy volunteers and IBS patients (Fig. 4B). IL-10, IL-13, and IL-4 were detected in 95%, 45%, 2% of the sera from healthy volunteers compared to 4%, 77%, 7%, respectively for ID-IBS and 0%, 25%, 6%, respectively for PI-IBS. On the other hand, fecal and sera concentrations of anti-inflammatory cytokine, IL-10 were significantly higher in the healthy volunteers than that of the IBS patients. Together, these findings suggest an increased secretion of pro-inflammatory cytokines and decreased anti-inflammatory cytokine IL-10 in IBS. This is consistent with increased chemotactic chemokines, which recruits inflammatory cells to the intestinal lumen.

**Figure 6:**
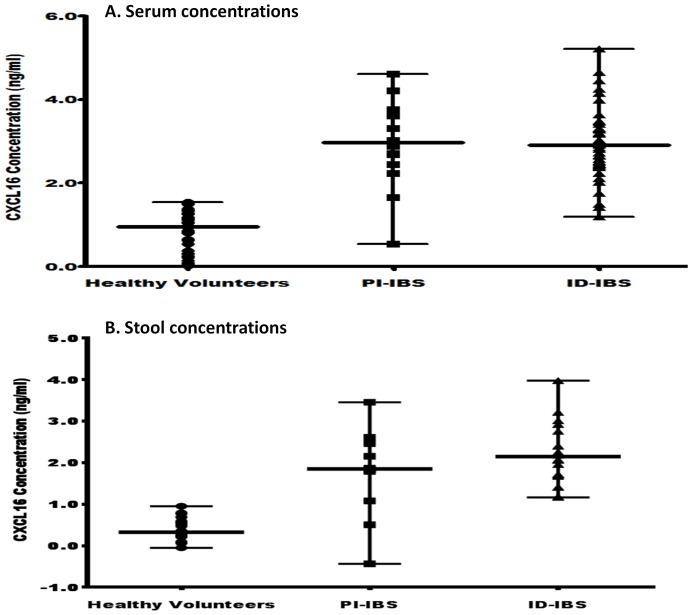
The sera and stools of IBS patients indicate higher levels of the chemokine CXCL16 than that of healthy volunteers. Sera and stools from IBS patients and healthy volunteers were evaluated for CXCL16 concentration using quantitative ELISA (R&D Systems, Minneapolis, MN). Mann Whitney test showed significant difference between the medians of the healthy volunteers versus ID-IBS (P<0.0001) and PI-IBS (P<0.0001). The medians of ID-IBS versus PI-IBS were not significantly different (P = 0.6219). Error bars indicate median values with range.

Chemokine CXCL16, a microbially-regulated factor that modulates the quantities and function of NTK cells in the gastrointestinal tract, is highly expressed in human epithelial cells and increased in inflammation [18], [19], [20]. To investigate whether CXCL16 may play a role in IBS, sera and fecal concentrations were analyzed by quantitative ELISA (R&D Systems, Minneapolis, MN). The concentration of CXCL16 was significantly higher in both sera and stools of ID-IBS and PI-IBS patients compared to that of the healthy volunteers (Fig. 6). However, no significant differences were found between ID-IBS and PI-IBS (p = 0.5605). These results underscore the importance of this chemokine in IBS and suggest inflammation as an underlining mechanism in the pathogenesis of IBS.

## Discussion

Irritable bowel syndrome is one of the most frequently diagnosed disorders in the general population, affecting up to 20% of adolescents and adults in the Western countries [2], [3], [4], [21]. About 28% of patients seen in gastroenterology practice and 12% seen in primary care practice are diagnosed with IBS. This syndrome poses an enormous socioeconomic burden, substantially impairs the quality of life, and increases healthcare costs. Currently, there is no biomarker for IBS. In this study, we evaluated sera and stools of IBS patients and healthy volunteers with the goal to uncover major biomarkers that are associated with IBS and to provide insight into the immunopathogenesis of ID-IBS and PI-IBS. We demonstrate for the first time that the levels of monocytes chemotactic protein-1 (MCP-1), macrophage inflammatory protein-1β (MIP-1β), CXCL16 are elevated in IBS patients. However, the levels of these chemokines were not significantly different between ID-IBS and PI-IBS, suggesting that these two forms of IBS exhibit similar phenotypes despite their different etiological origins.

Chemokines are potent regulators of recruitment and migration of leukocytes to inflammatory sites [22]. These proteins are involved in a variety of inflammatory and immune responses, acting primarily as chemoattractants and activators of leukocytes. MCP-1 and MIP-1β are members of the C-C subfamily of chemokines [23], [24], [25]. MCP-1 is derived from mononuclear cells, fibroblasts, endothelial cells, smooth muscle cells, and epithelial cells. MCP-1 is a potent chemoattractant and mainly act on monocytes/macrophages by regulating the expression of adhesion molecules and cytokines in these cells [26], [27], [28], [29]. MCP-1 recruits monocytes/macrophages to inflammatory sites in various pathological conditions such as interstitial lung disease, atherosclerosis, rheumatoid arthritis, and glomerulonephritis [24], [30]. MIP-1β is secreted by activated T cells, B cells, monocytes, and mast cells and just as found in other C-C chemokines, MIP-1β chemoattractants monocytes and T cells [22]. These reported biological roles of MCP-1 and MIP-1β are consistent with our findings that these chemokines are elevated in IBS patients. One of the characteristics of IBS is inflammation. Thus, high levels of these pro-inflammatory chemokines highlight the importance and extent of inflammation in IBS.

Additionally, the concentrations of pro-inflammatory cytokines including IFN-γ, IL-1β, and TNF-α in both serum and fecal samples were higher in IBS patients than the healthy volunteers. However, the fecal and serum concentrations of anti-inflammatory cytokine IL-10 was significantly lower in the IBS groups. These results are consistent with previous reports of the levels of these cytokines in sera and mucosal biopsy samples of patients with IBS [31], [32], [33], [34], [35], [36], [37], [38].

We have also demonstrated for the first time that chemokine CXCL16 is significantly elevated in IBS patients. Compromised barrier function of the gut and aberrant immune response to intestinal pathogens are the cornerstones in the pathogenesis of inflammatory bowel disease. CXCL16 shares pattern recognition receptor functions necessary for adhesion and phagocytosis of bacterial products, with the properties of an adhesion molecule and inflammatory chemokine [39], [40], [41]. CXCL16 expression has been associated with a number of human inflammatory diseases [42], [43]. Intestinal microbiota may stimulate these chemotactic chemokines through release of short chain fatty acids [44]. Elevated levels of “C chemokines” (CXCL16) and “CC chemokines” (MCP-1 and MIP-1β) in IBS patients suggest that these small signaling proteins, which induce the chemotaxis of monocytes, macrophages, mast cells, T lymphocytes, eosinophils, and neutrophils to sites of inflammation may play a role in the pathophysiology of IBS.

One of the limitations of this study is the small sample size (44 ID-IBS, 16 PI-IBS, and 40 healthy volunteers). As part of a future study, we plan to enroll a larger number of subjects to confirm these findings and to elucidate the exact role of these chemokines in the pathogenesis of IBS.

The data obtained from sera correlated with that from stool and this indicate that fecal samples can be used in patients with IBS to study immune reactivity. Our results demonstrate that both PI-IBS and ID-IBS show similar immune alterations and suggest that the pathogenesis of these two forms of IBS is similar despite having apparently different inciting events. A biomarker is needed to identify patients with IBS since there is no gold standard for IBS diagnosis. Some authors have suggested that low serum IL-10 could be a biomarker to identify cases of IBS. Perhaps the ratio of a pro-inflammatory cytokine or chemokine divided by IL-10 levels using sera or fecal samples would serve as a useful biomarker.

## Supporting Information

File S1 **Contains Supplement S1 and Supplement S2: S1.** A list of biomarkers that were evaluated and their reported roles in inflammation and immune response. **S2**. Serum (A) and fecal (B) concentrations (in pg/ml) of key inflammatory markers detected in IBS patients and healthy volunteers.
(PDF)
