# Supplementary material for: Chemotactic Chemokines Are Important in the Pathogenesis of Irritable Bowel Syndrome
Source: PLoS One. 2014 Mar 25;9(3):e93144. doi: 10.1371/journal.pone.0093144 (PMC3965506; doi:10.1371/journal.pone.0093144)
Supplement: File S1 — Contains Supplement S1 and Supplement S2: S1. A list of biomarkers that were evaluated and their reported roles in inflammation and immune response. S2. Serum (A) and fecal (B) concentrations (in pg/ml) of key inflammatory markers detected in IBS patients and healthy volunteers. (PDF) [file pone.0093144.s001.pdf]

Supplemental Materials

S1. A list of biomarkers that were evaluated and their reported roles in inflammation and immune response.

MARKER	ROLE IN INFLAMMATION
**C5a**	Chemoattractant, activates phagocytes, anaphylotoxin, aggregates neutrophils and platelets [1]
**CD40L**	Mediates B cell proliferation and Ig class switching
**G-CSF**	Stimulates neutrophil development and granulocyte release [2,3]
**GM-CSF**	Stimulates polymorphonuclear cells, differentiation of hematopoietic cells [2,3]
**GROα**	Neutrophilic chemoattraction and infiltration [3,4]
**I-309**	Chemotaxis of monocytes, intracellular Ca^{2+} mobilization [4,5]
**sICAM-1**	Adhesion and transmigration of leukocytes [6]
**IFN-γ**	Enhances MHC class I and class II expression, macrophage activation [2,3,7]
**IL-1α & IL-1β**	Induces CD4 T cell differentiation, proliferation of mature B cells, PGE_2 synthesis, mast cell histamine release, induces IL-2 expression [2,3]
**IL-1ra**	Inhibits IL-1 by competitive binding to IL-1 receptor [2,3,7]
**IL-2**	Activates T cells, NK cells, and B cells, class II MHC expression [2,3,7]
**IL-4**	Stimulates B/T-cell proliferation, suppresses development of Th1 cells, promotes Ig class switching, antagonizes IFN-γ expression, anti-inflammatory, suppresses macrophage cytotoxicity [2,3,7]
**IL-5**	Eosinophil differentiation and activation, Ig class switching, B cell proliferation, anti-inflammatory [2,3,7]
**IL-6**	Stimulates acute phase protein synthesis, inhibits pro-inflammatory cytokines, promotes activation and differentiation of plasma cells and T-cells [2,8]
**IL-8**	Chemoattracts leukocytes to inflammation sites, upregulates adhesion molecules expression and adherence of leukocytes to endothelial cells [3,4]
**IL-10**	Inhibits IFN-γ production by NK cells and activated T-cells, dampens Th1 response, inhibits synthesis of IL-1β and NFΚB nuclear translocation [3,7]
**IL-12p70**	Enhances cytotoxic T-cell and NK cell proliferation and cytotoxicity, increases IFN-γ production in T cells and NK cells, inhibits IgE release from B cells [3,7]

**IL-13**	Inhibits inflammatory cytokine production, induces Ig class switching to IgG_4 and IgE, attenuates macrophage activation [3,7]
**IL-16**	Chemotaxis of CD4 T lymphocytes [2,9]
**IL-17**	Stimulates IL-6, IL-8, and ICAM expression [2,10]
**IL-17E**	Induces pro-inflammatory cytokine response, increase serum IgE and IgG_1 production, NFKB activation [11]
**IL-23**	Activation of CD4 memory T-cells, induces IL-17 and IL-6, pro-inflammatory [12]
**IL-27**	Stimulates proliferation of naïve CD4 T-cells, Ig class switching, induces T-bet and IL-12R expression in naïve T cells [13]
**IL-32α**	Activation of P38 MAPK and NFKB pathways, induces secretion of pro-inflammatory cytokines such as TNF-α and MIP-2 [14]
**IP-10**	T-cells, NK cells, and monocytes chemoattractant, promotes T-cell adherence to endothelial cells [2,4]
**I-TAC**	Th1 inflammation, recruits activated T-cells to site of inflammation [15]
**MCP-1**	Chemotactic activation and migration of leukocytes to site of infection, stimulates histamine release [2,3,7]
**MIF**	Activates production of macrophages, induces TNF-α production [16]
**MIP-1α**	Chemotactic activation and migration of leukocytes to sites of inflammation, inhibits proliferation of early hematopoietic stem cells [2,3,7]
**MIP-1β**	Chemotactic activation and migration of lymphocytes to sites of inflammation [2,3,7]
**PAI-1**	Extracellular matrix proteolysis, cellular adhesion and migration, inhibitory role in fibrinolytic system [17]
**RANTES**	Chemotactic activation and migration of leukocytes to sites of inflammation, stimulates histamine release [2,3,7]
**SDF-1**	Chemoattractant for lymphocytes, progenitor cell development in bone marrow [4]
**TNF-α**	Stimulation of PGE_2, stimulates acute and chronic inflammation, induces apoptosis, induction of acute phase reactant proteins [2,18]
**sTREM-1**	Release of IL-8, MCP-1, TNF-α, induces neutrophil degranulation [19]

S2. Serum (A) and fecal (B) concentrations (in pg/ml) of key inflammatory markers detected in IBS patients and healthy volunteers.

A. Serum concentrations

INFLAMMATORY MARKER	HEALTHY CONTROL N= 40			IDIOPATHIC-IBS N= 44			POST-INFECTIOUS-IBS N= 16		
	MEAN	MEDIAN	RANGE	MEAN	MEDIAN	RANGE	MEAN	MEDIAN	RANGE
MCP-1	295±17.5	283.6	102-576.2	454±30.1	390.6	114-955.0	467±61.5	421.3	212.3-1170
MIP-1β	47±3.6	45.2	1-111.1	220±22.8	213.3	28-579.5	216.2±38	173.3	41-462.3
IFN-γ	29±1.7	32.4	0.1-47.5	49±5.6	37.2	7-184.3	64±14.7	40.6	30-238.9
TNF-α	22.8±0.9	24.5	0.2-30.4	34±3.9	27.0	1-129.1	36±5.6	27.0	23-86.1
IL-1β	5.3±0.3	5.4	0.02-10.3	9.8±1.5	5.8	4-53.1	10.2±2.5	6.0	4.7-36.6
IL-10	11.3±0.8	13.1	4.4-20.1	5.1±0.3	5.3	0.1-10.1	6.5±0.6	5.6	3.6-13.2
CXCL16	873±0.07	947	11-1544	2915±240	2966	543-4608	2908±126	2830	1196-5213

B. Fecal concentrations

INFLAMMATORY MARKER	HEALTHY CONTROL N= 20			IDIOPATHIC-IBS N= 20			POST-INFECTIOUS-IBS N= 10		
	MEAN	MEDIAN	RANGE	MEAN	MEDIAN	RANGE	MEAN	MEDIAN	RANGE
MCP-1	149.6±18	137.2	25-361.6	305±24	289.2	113.9-464	235.9±26	222.5	112-369
MIP-1β	85.6±10	74.0	20-149.8	170.4±11	178.0	88-312.8	145.9±10	137.8	100-199.7
IFN-γ	20.2±1.4	22.0	40.3-31.7	41.7±7.3	28.9	24.5-156	35.9±5.7	29.6	21-73.6
TNF-α	14.8±1.9	18.4	1.4-24.0	24±1.7	20.5	18.2-48.0	26.5±5.7	20.2	16.1-77.1
IL-1β	5.3±0.4	4.9	2.1-9.4	7.4±1.5	4.5	3-28.9	7.7±1.6	4.9	3.7-24.8
IL-10	10.1±1.6	7.6	4.5-37.9	4.6±0.6	5.1	0.02-12.5	4.8±1.1	5.1	0.02-15.7
CXCL16	405±64.5	327	48-952	2150±163	2066	1165-3978	1733±351	1852	1200-5200

References

1. Guo RF, Ward PA (2005) Role of C5a in inflammatory responses. Annu Rev Immunol 23: 821-852.

2. Feghali CA, Wright TM (1997) Cytokines in acute and chronic inflammation. Front Biosci 2: d12-26.

3. Liles WC, Van Voorhis WC (1995) Review: nomenclature and biologic significance of cytokines involved in inflammation and the host immune response. J Infect Dis 172: 1573-1580.

4. Luster AD (1998) Chemokines--chemotactic cytokines that mediate inflammation. N Engl J Med 338: 436-445.

5. Haque NS, Zhang X, French DL, Li J, Poon M, et al. (2000) CC chemokine I-309 is the principal monocyte chemoattractant induced by apolipoprotein(a) in human vascular endothelial cells. Circulation 102: 786-792.

6. Ridker PM, Hennekens CH, Roitman-Johnson B, Stampfer MJ, Allen J (1998) Plasma concentration of soluble intercellular adhesion molecule 1 and risks of future myocardial infarction in apparently healthy men. Lancet 351: 88-92.

7. Opal SM, DePalo VA (2000) Anti-inflammatory cytokines. Chest 117: 1162-1172.

8. Cameron MJaK, D. J. (2000) Cytokines, Chemokines and Their Receptors. Madame Curie Bioscience Database. Austin: Landes Bioscience.

9. Rumsaeng V, Cruikshank WW, Foster B, Prussin C, Kirshenbaum AS, et al. (1997) Human mast cells produce the CD4+ T lymphocyte chemoattractant factor, IL-16. J Immunol 159: 2904-2910.

10. Fossiez F, Banchereau J, Murray R, Van Kooten C, Garrone P, et al. (1998) Interleukin-17. Int Rev Immunol 16: 541-551.

11. Pan G, French D, Mao W, Maruoka M, Risser P, et al. (2001) Forced expression of murine IL-17E induces growth retardation, jaundice, a Th2-biased response, and multiorgan inflammation in mice. J Immunol 167: 6559-6567.

12. Yen D, Cheung J, Scheerens H, Poulet F, McClanahan T, et al. (2006) IL-23 is essential for T cell-mediated colitis and promotes inflammation via IL-17 and IL-6. J Clin Invest 116: 1310-1316.

13. Liu L, Wang S, Shan B, Shao L, Sato A, et al. (2008) IL-27-mediated activation of natural killer cells and inflammation produced antitumour effects for human oesophageal carcinoma cells. Scand J Immunol 68: 22-29.

14. Moschen AR, Fritz T, Clouston AD, Rebhan I, Bauhofer O, et al. (2011) Interleukin-32: a new proinflammatory cytokine involved in hepatitis C virus-related liver inflammation and fibrosis. Hepatol 53: 1819-1829.

15. Helbig KJ, Ruszkiewicz A, Semendric L, Harley HA, McColl SR, et al. (2004) Expression of the CXCR3 ligand I-TAC by hepatocytes in chronic hepatitis C and its correlation with hepatic inflammation. Hepatol 39: 1220-1229.

16. Calandra T, Bernhagen J, Mitchell RA, Bucala R (1994) The macrophage is an important and previously unrecognized source of macrophage migration inhibitory factor. J Exp Med 179: 1895-1902.

17. Renckens R, Roelofs JJ, de Waard V, Florquin S, Lijnen HR, et al. (2005) The role of plasminogen activator inhibitor type 1 in the inflammatory response to local tissue injury. J Thromb Haemost 3: 1018-1025.

18. Idriss HT, Naismith JH (2000) TNF alpha and the TNF receptor superfamily: structure-function relationship(s). Microsc Res Tech 50: 184-195.

19. Radsak MP, Salih HR, Rammensee HG, Schild H (2004) Triggering receptor expressed on myeloid cells-1 in neutrophil inflammatory responses: differential regulation of activation and survival. J Immunol 172: 4956-4963.
